# (2*R*,3*S*)-Methyl 2-hy­droxy-3-(4-methyl­benzene­sulfonamido)-3-phenyl­propano­ate

**DOI:** 10.1107/S160053681004780X

**Published:** 2010-11-24

**Authors:** Mohamed I. Fadlalla, Holger B. Friedrich, Glenn E. M. Maguire, Bernard Omondi

**Affiliations:** a School of Chemistry, University of KwaZulu-Natal, Westville Campus, Private Bag X54001, Durban 4000, South Africa; b Research Centre for Synthesis and Catalysis, Department of Chemistry, University of Johannesburg, PO Box 524 Auckland Park, Johannesburg 2006, South Africa

## Abstract

In the title mol­ecule, C_17_H_19_NO_5_S, the *p*-tolyl ring is oriented approximately parallel to the phenyl ring [dihedral angle = 17.2 (1)°], resulting in an intra­molecular π–π inter­ation [centroid–centroid distance = 3.184 (10) Å]. In the crystal, mol­ecules are linked through O—H⋯O and C—H⋯O hydrogen bonds, forming hydrogen-bonded sheets lying diagonally across the *ac* face.

## Related literature

For related structures of β-amino alcohols, see: Bergmeier (2000 ▶); Krzeminski & Wojtczak (2005 ▶). For related structures of tosyl­amino compounds, see: Coote *et al.* (2008 ▶); Liu *et al.* (2005 ▶); Chinnakali *et al.* (2007 ▶); Nan & Xing (2006 ▶); Fadlalla *et al.* (2010 ▶); Zhao *et al.* (2005 ▶). For the synthesis of the title compound, see: Naicker *et al.* (2008 ▶); Govender *et al.* (2003 ▶). For the use of β-amino alcohols in the synthesis of chiral ligands for asymmetric catalysis, see: Bodkin & McLeod (2002 ▶); Lohray *et al.* (2002 ▶).
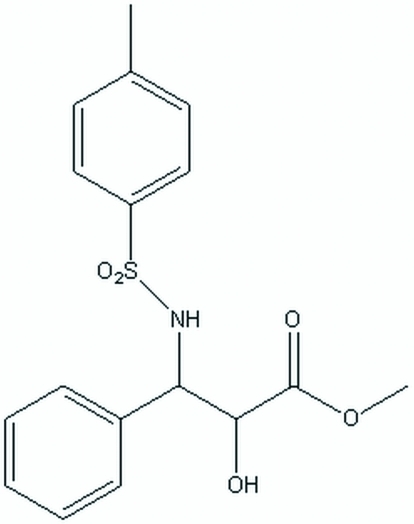


## Experimental

### 

#### Crystal data

C_17_H_19_NO_5_S
*M* *_r_* = 349.39
Monoclinic, 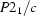

*a* = 10.4053 (8) Å
*b* = 5.4655 (4) Å
*c* = 29.3768 (19) Å
β = 105.386 (3)°
*V* = 1610.8 (2) Å^3^
*Z* = 4
Mo *K*α radiation
μ = 0.23 mm^−1^
*T* = 100 K
0.13 × 0.11 × 0.09 mm

#### Data collection

Bruker X8 APEXII 4K Kappa CCD diffractometer
Absorption correction: multi-scan (*SADABS*; Bruker, 2007 ▶) *T*_min_ = 0.971, *T*_max_ = 0.980
17784 measured reflections
4016 independent reflections
3212 reflections with *I* > 2σ(*I*)
*R*_int_ = 0.038

#### Refinement

*R*[*F* ^2^ > 2σ(*F* ^2^)] = 0.04
*wR*(*F* ^2^) = 0.100
*S* = 1.00
4016 reflections
219 parameters
H-atom parameters constrained
Δρ_max_ = 0.67 e Å^−3^
Δρ_min_ = −0.65 e Å^−3^

### 

Data collection: *APEX2* (Bruker, 2007 ▶); cell refinement: *SAINT-Plus* (Bruker, 2007 ▶); data reduction: *SAINT-Plus* and *XPREP* (Bruker, 2007 ▶); program(s) used to solve structure: *SHELXS97* (Sheldrick, 2008 ▶); program(s) used to refine structure: *SHELXL97* (Sheldrick, 2008 ▶); molecular graphics: *DIAMOND* (Brandenburg & Putz, 2005 ▶), *ORTEP-3* (Farrugia, 1999 ▶); software used to prepare material for publication: *WinGX* (Farrugia, 1999 ▶).

## Supplementary Material

Crystal structure: contains datablocks global, I. DOI: 10.1107/S160053681004780X/fl2324sup1.cif

Structure factors: contains datablocks I. DOI: 10.1107/S160053681004780X/fl2324Isup2.hkl

Additional supplementary materials:  crystallographic information; 3D view; checkCIF report

## Figures and Tables

**Table 1:** Hydrogen-bond geometry (Å, °)

*D*—H⋯*A*	*D*—H	H⋯*A*	*D*⋯*A*	*D*—H⋯*A*
O3—H3*A*⋯O2^i^	0.84	2.50	3.270 (2)	152
C1—H1*C*⋯O1^ii^	0.98	2.52	3.392 (2)	149
C4—H4⋯O3^iii^	1.00	2.50	3.484 (2)	166
C1—H1*C*⋯O1^ii^	0.98	2.52	3.392 (2)	149

Symmetry codes: (i) 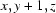; (ii) 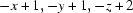; (iii) 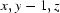.

**Fig. 1.**
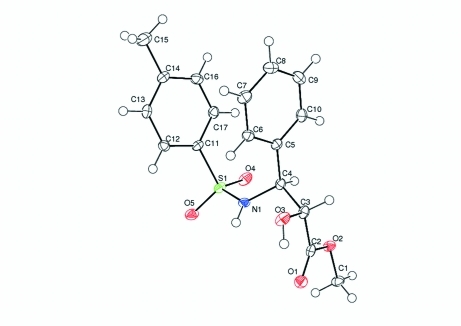
View of (I) (50% probability displacement ellipsoids) with H atoms presented as small spheres of arbitrary radii.

**Fig. 2.**
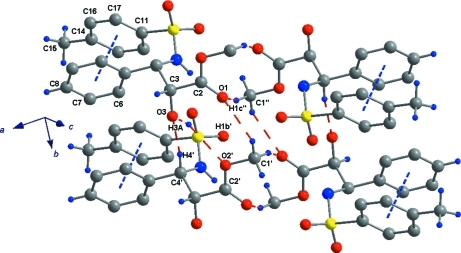
O—H···O and C—H···O hydrogen bond interactions in the crystal structure of (I). [Symmetry operators: (i) = x, 1 + y, z; (ii) = 1 - x, 1 - y, 2 - z]

**Fig. 3.**
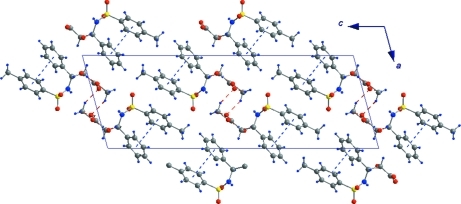
Sheets of O—H···O and C—H···O hydrogen bonded molecules alligned diagonally across the ac face.

## supplementary crystallographic information

### Comment

Vicinal amino alcohols (beta-amino alcohol) are a common structural component in many naturally occuring and biologically active compounds. Furthermore, beta-amino alcohols are used in the synthesis of chiral ligands for asymmetric catalysis (Lohray *et al.*, 2002, Bodkin & McLeod, 2002). As part of investigating a new synthetic route to these molecules, we report the crystal structure of the title compound (I) (Fig. 1) whose synthesis produces diastereomers which are separable using chromatography with the stable one being the one with the chirality as R at C3 and S at C4.

The molecular structure of the title compound, C_17_H_19_NO_5_S (I), is similar to that of *trans*-methyl 2-hydroxy-3-(*p*-fluoro)phenyl-3'- (*N*-tosyl amino)propanoate (Zhao *et al.*, 2005). The crystal structure is characterized by a number of intra- and inter- molecular interactions. An O-H···O and three C-H···O hydrogen bonds (Table 1) stabilize the crystal structure forming hydrogen bonded sheets that run along the b axis. In addition the p-tolyl and phenyl rings are in close proximity leading to a π–π interaction (Cg1···Cg2 = 3.8149 (10) Å) (Fig. 2). The hydrogen bonded sheets of molecules are alligned along the crystallographic *ac* face (Fig 3). It is worth mentioning that the H on the N atom does not contribute to a hydrogen bond as their is no acceptor in close proximity.

### Experimental

The title compound was obtained through a modified literature method (Naicker *et al.*, 2008, Govender *et al.*, 2003). To a nitrogen saturated Schlenk tube 6 ml of a mixture of acetonitrile and water (1:1 *v*/*v*), methyl cinnamate (0.0775 g, 0.478 mmol), chloramine-T (0.2173 g, 0.956 mmol), hydrotalcite-like catalyst (0.03 g) were added in that order. The catalyst was gravity filtered off after 24 h. The reaction mixture was then washed with sodium sulfite (1 g in 20 ml of de-ionized water) followed by 15 ml of ethyl acetate. The aqueous layer was separated from the organic layer and further washed by 3x 15 ml of ethyl acetate. The solvent of the combined organic mixture was removed *in vacuo*. The resulting crude product was purified by preparative high preasure liquid chromatography to yield the title compound, (I), as a white solid. Crystals of I were obtained by slow evaporation of a solution of acetonitrile and water (1:1 *v*/*v*) at room temperature (m.p. 413–418 K). Spectroscopic data: ^1^H NMR (400 MHz, CDCl_3_, δ. p.p.m.): = 2.3 (s, 3H), 3.3 (d, 1H), 3.7 (s, 3H), 4.3 (d, 1H), 4.8 (dd, 1H), 5.6 (d, 1H), 7.0–7.1 (m, Ar), 7.5 (m, Ar). ^13^C NMR (400 MHz, CDCl_3_, δ. p.p.m.): =21.4 (s, 1 C), 53.2 (s, 1 C), 58.9 (s, 1 C), 74.2 (s, 1 C), 126.8 (s, 2 C), 126.9 (s, 2 C), 127.8 (s, 2 C), 128.4 (s, 2 C), 129.2 (s, 1 C), 137.4 (s, 1 C), 137.5 (s, 1 C), 143.1 (s, 1 C), 172.4 (s, 1 C). MS m/*z*-[fragment]-(%): 372.1 (*M* + Na^+^) calculated = 372.1 for C_17_H_19_NO_5_SNa^+^. FT—IR (cm^1^): = 3477(*m*), (OH), 3139(*m*), (NH), 2967(w), 2882(w), 1598(w), (ar), 1738(*m*), (C=O), 1056(*m*), (S=O).

### Refinement

The methyl, methine and aromatic H atoms were placed in geometrically idealized positions and constrained to ride on their parent atoms, with C—H = 0.95 Å and *U*_iso_(H) = 1.2*U*_eq_(C) for aromatic, C—H = 0.98 Å and *U*_iso_(H) = 1.2*U*_eq_(C) for CH_3_, C—H = 1.00 Å and *U*_iso_(H) = 1.2*U*_eq_(C) for CH. N—H = 0.88 Å and *U*_iso_(H) = 1.2*U*_eq_(N) for N—H and O—H = 0.84 Å and *U*_iso_(H) = 1.5*U*_eq_(O).

### Crystal data

**Table:**

C_17_H_19_NO_5_S	*F*(000) = 736
*M**_r_* = 349.39	*D*_x_ = 1.441 Mg m^−^^3^
Monoclinic, *P*2_1_/*c*	Mo *K*α radiation, λ = 0.71073 Å
Hall symbol: -P 2ybc	Cell parameters from 18868 reflections
*a* = 10.4053 (8) Å	θ = 2.0–28.4°
*b* = 5.4655 (4) Å	µ = 0.23 mm^−^^1^
*c* = 29.3768 (19) Å	*T* = 100 K
β = 105.386 (3)°	Block, colourless
*V* = 1610.8 (2) Å^3^	0.13 × 0.11 × 0.09 mm
*Z* = 4	

### Data collection

**Table:**

Bruker X8 APEXII 4K Kappa CCD diffractometer	3212 reflections with *I* > 2σ(*I*)
graphite	*R*_int_ = 0.038
φ and ω scans	θ_max_ = 28.4°, θ_min_ = 2.0°
Absorption correction: multi-scan (*SADABS*; Bruker, 2007)	*h* = −13→13
*T*_min_ = 0.971, *T*_max_ = 0.980	*k* = −4→7
17784 measured reflections	*l* = −39→39
4016 independent reflections	

### Refinement

**Table:**

Refinement on *F*^2^	Primary atom site location: structure-invariant direct methods
Least-squares matrix: full	Secondary atom site location: difference Fourier map
*R*[*F*^2^ > 2σ(*F*^2^)] = 0.04	Hydrogen site location: inferred from neighbouring sites
*wR*(*F*^2^) = 0.100	H-atom parameters constrained
*S* = 1.00	*w* = 1/[σ^2^(*F*_o_^2^) + (0.0409*P*)^2^ + 1.5018*P*] where *P* = (*F*_o_^2^ + 2*F*_c_^2^)/3
4016 reflections	(Δ/σ)_max_ = 0.034
219 parameters	Δρ_max_ = 0.67 e Å^−^^3^
0 restraints	Δρ_min_ = −0.65 e Å^−^^3^

### Special details

**Table:**

Experimental. The intensity data was collected on a Bruker X8 Apex 4 K CCD diffractometer using an exposure time of 15 sec/per frame. A total of 1480 frames were collected with a frame width of 0.5° covering upto θ = 28.41° with 99.8% completeness accomplished.
Geometry. All e.s.d.'s (except the e.s.d. in the dihedral angle between two l.s. planes) are estimated using the full covariance matrix. The cell e.s.d.'s are taken into account individually in the estimation of e.s.d.'s in distances, angles and torsion angles; correlations between e.s.d.'s in cell parameters are only used when they are defined by crystal symmetry. An approximate (isotropic) treatment of cell e.s.d.'s is used for estimating e.s.d.'s involving l.s. planes.
Refinement. Refinement of *F*^2^ against ALL reflections. The weighted *R*-factor *wR* and goodness of fit *S* are based on *F*^2^, conventional *R*-factors *R* are based on *F*, with *F* set to zero for negative *F*^2^. The threshold expression of *F*^2^ > σ(*F*^2^) is used only for calculating *R*-factors(gt) *etc*. and is not relevant to the choice of reflections for refinement. *R*-factors based on *F*^2^ are statistically about twice as large as those based on *F*, and *R*- factors based on ALL data will be even larger. >>> The Following Model and Quality ALERTS were generated - (Acta-Mode) <<< Format: alert-number_ALERT_alert-type_alert-level text 960_ALERT_3_G Number of Intensities with *I*. LT. - 2*sig(*I*).. 1 793_ALERT_4_G The Model has Chirality at C3 (Verify) ···. *R* 793_ALERT_4_G The Model has Chirality at C4 (Verify) ···. *S* The chirality is verified and correct.

### Fractional atomic coordinates and isotropic or equivalent isotropic displacement parameters (Å^2^)

**Table:**

	*x*	*y*	*z*	*U*_iso_*/*U*_eq_	
C1	0.60326 (17)	0.1396 (3)	1.05597 (6)	0.0179 (3)	
H1A	0.6289	0.2404	1.0845	0.027*	
H1B	0.6083	−0.0337	1.0648	0.027*	
H1C	0.5118	0.1798	1.0383	0.027*	
C2	0.70299 (16)	0.4237 (3)	1.01583 (6)	0.0151 (3)	
C3	0.80464 (16)	0.4702 (3)	0.98772 (6)	0.0154 (3)	
H3	0.8947	0.4182	1.0071	0.018*	
C4	0.77208 (15)	0.3308 (3)	0.94058 (6)	0.0140 (3)	
H4	0.7672	0.1526	0.9476	0.017*	
C5	0.88195 (16)	0.3659 (3)	0.91578 (6)	0.0144 (3)	
C6	0.88826 (16)	0.5749 (3)	0.88948 (6)	0.0166 (3)	
H6	0.8229	0.6993	0.8868	0.02*	
C7	0.98912 (17)	0.6037 (3)	0.86702 (6)	0.0203 (4)	
H7	0.992	0.7462	0.8488	0.024*	
C8	1.08600 (17)	0.4233 (3)	0.87122 (6)	0.0216 (4)	
H8	1.1555	0.4429	0.8561	0.026*	
C9	1.08063 (17)	0.2154 (3)	0.89751 (6)	0.0212 (4)	
H9	1.1467	0.0921	0.9004	0.025*	
C10	0.97868 (16)	0.1857 (3)	0.91979 (6)	0.0175 (3)	
H10	0.9754	0.0423	0.9377	0.021*	
C11	0.62837 (16)	0.3006 (3)	0.82070 (6)	0.0149 (3)	
C12	0.59763 (16)	0.5127 (3)	0.79368 (6)	0.0160 (3)	
H12	0.536	0.628	0.7998	0.019*	
C13	0.65878 (16)	0.5527 (3)	0.75750 (6)	0.0175 (3)	
H13	0.6381	0.6965	0.7388	0.021*	
C14	0.74982 (17)	0.3851 (3)	0.74828 (6)	0.0188 (4)	
C15	0.8146 (2)	0.4311 (4)	0.70873 (7)	0.0325 (5)	
H15A	0.8962	0.3336	0.714	0.049*	
H15B	0.8367	0.6052	0.708	0.049*	
H15C	0.7529	0.3846	0.6786	0.049*	
C16	0.77809 (17)	0.1746 (3)	0.77556 (6)	0.0201 (4)	
H16	0.8393	0.0585	0.7694	0.024*	
C17	0.71818 (17)	0.1312 (3)	0.81180 (6)	0.0178 (3)	
H17	0.7385	−0.0131	0.8303	0.021*	
N1	0.63870 (13)	0.4102 (3)	0.91306 (5)	0.0153 (3)	
H1	0.6036	0.5446	0.9211	0.018*	
O1	0.64240 (12)	0.5874 (2)	1.02804 (4)	0.0191 (3)	
O2	0.69409 (12)	0.1881 (2)	1.02642 (4)	0.0170 (3)	
O3	0.80780 (12)	0.7244 (2)	0.97843 (4)	0.0196 (3)	
H3A	0.7704	0.8013	0.9961	0.029*	
O4	0.57588 (13)	−0.0023 (2)	0.88103 (4)	0.0214 (3)	
O5	0.42425 (12)	0.3532 (3)	0.85460 (4)	0.0235 (3)	
S1	0.55584 (4)	0.25129 (8)	0.867939 (14)	0.01596 (11)	

### Atomic displacement parameters (Å^2^)

**Table:**

	*U*^11^	*U*^22^	*U*^33^	*U*^12^	*U*^13^	*U*^23^
C1	0.0213 (8)	0.0183 (8)	0.0179 (8)	0.0000 (6)	0.0115 (7)	0.0030 (7)
C2	0.0173 (7)	0.0162 (8)	0.0101 (7)	−0.0019 (6)	0.0009 (6)	0.0002 (6)
C3	0.0193 (8)	0.0137 (8)	0.0133 (7)	−0.0030 (6)	0.0043 (6)	0.0013 (6)
C4	0.0158 (7)	0.0133 (8)	0.0130 (7)	−0.0006 (6)	0.0040 (6)	0.0016 (6)
C5	0.0155 (7)	0.0151 (8)	0.0124 (7)	−0.0020 (6)	0.0032 (6)	−0.0023 (6)
C6	0.0189 (8)	0.0153 (8)	0.0164 (8)	0.0017 (6)	0.0062 (6)	0.0005 (7)
C7	0.0243 (9)	0.0187 (9)	0.0203 (9)	−0.0039 (7)	0.0104 (7)	0.0005 (7)
C8	0.0176 (8)	0.0262 (10)	0.0230 (9)	−0.0031 (7)	0.0086 (7)	−0.0063 (8)
C9	0.0165 (8)	0.0225 (9)	0.0231 (9)	0.0034 (7)	0.0030 (7)	−0.0044 (7)
C10	0.0188 (8)	0.0153 (8)	0.0168 (8)	0.0005 (6)	0.0020 (6)	0.0000 (7)
C11	0.0161 (7)	0.0180 (8)	0.0114 (7)	−0.0035 (6)	0.0048 (6)	−0.0021 (6)
C12	0.0161 (7)	0.0159 (8)	0.0158 (8)	0.0016 (6)	0.0041 (6)	−0.0017 (7)
C13	0.0196 (8)	0.0166 (8)	0.0148 (8)	−0.0006 (6)	0.0019 (6)	0.0031 (7)
C14	0.0215 (8)	0.0216 (9)	0.0147 (8)	−0.0012 (7)	0.0073 (6)	0.0000 (7)
C15	0.0410 (11)	0.0362 (12)	0.0271 (10)	0.0073 (9)	0.0212 (9)	0.0076 (9)
C16	0.0223 (8)	0.0191 (9)	0.0210 (9)	0.0046 (7)	0.0096 (7)	−0.0007 (7)
C17	0.0226 (8)	0.0144 (8)	0.0166 (8)	0.0014 (6)	0.0055 (7)	0.0013 (7)
N1	0.0153 (6)	0.0183 (7)	0.0128 (6)	0.0006 (5)	0.0048 (5)	−0.0032 (6)
O1	0.0237 (6)	0.0152 (6)	0.0191 (6)	0.0006 (5)	0.0066 (5)	−0.0009 (5)
O2	0.0226 (6)	0.0144 (6)	0.0171 (6)	0.0010 (5)	0.0108 (5)	0.0032 (5)
O3	0.0314 (7)	0.0125 (6)	0.0168 (6)	−0.0041 (5)	0.0097 (5)	−0.0001 (5)
O4	0.0298 (7)	0.0189 (6)	0.0175 (6)	−0.0092 (5)	0.0101 (5)	−0.0015 (5)
O5	0.0158 (6)	0.0371 (8)	0.0180 (6)	−0.0018 (5)	0.0052 (5)	−0.0040 (6)
S1	0.01589 (19)	0.0203 (2)	0.01270 (19)	−0.00410 (15)	0.00551 (14)	−0.00200 (16)

### Geometric parameters (Å, °)

**Table:**

C1—O2	1.4671 (19)	C9—H9	0.95
C1—H1A	0.98	C10—H10	0.95
C1—H1B	0.98	C11—C17	1.389 (2)
C1—H1C	0.98	C11—C12	1.393 (2)
C2—O1	1.203 (2)	C11—S1	1.7675 (16)
C2—O2	1.334 (2)	C12—C13	1.393 (2)
C2—C3	1.526 (2)	C12—H12	0.95
C3—O3	1.418 (2)	C13—C14	1.395 (2)
C3—C4	1.537 (2)	C13—H13	0.95
C3—H3	1	C14—C16	1.388 (3)
C4—N1	1.474 (2)	C14—C15	1.511 (2)
C4—C5	1.522 (2)	C15—H15A	0.98
C4—H4	1	C15—H15B	0.98
C5—C6	1.390 (2)	C15—H15C	0.98
C5—C10	1.390 (2)	C16—C17	1.390 (2)
C6—C7	1.389 (2)	C16—H16	0.95
C6—H6	0.95	C17—H17	0.95
C7—C8	1.392 (3)	N1—S1	1.6274 (14)
C7—H7	0.95	N1—H1	0.88
C8—C9	1.383 (3)	O3—H3A	0.8401
C8—H8	0.95	O4—S1	1.4387 (14)
C9—C10	1.396 (2)	O5—S1	1.4333 (13)
			
O2—C1—H1A	109.5	C9—C10—H10	120
O2—C1—H1B	109.5	C5—C10—H10	120
H1A—C1—H1B	109.5	C17—C11—C12	120.78 (15)
O2—C1—H1C	109.5	C17—C11—S1	119.66 (13)
H1A—C1—H1C	109.5	C12—C11—S1	119.54 (13)
H1B—C1—H1C	109.5	C13—C12—C11	118.88 (15)
O1—C2—O2	125.07 (15)	C13—C12—H12	120.6
O1—C2—C3	122.04 (15)	C11—C12—H12	120.6
O2—C2—C3	112.85 (14)	C12—C13—C14	121.06 (16)
O3—C3—C2	108.80 (13)	C12—C13—H13	119.5
O3—C3—C4	108.86 (13)	C14—C13—H13	119.5
C2—C3—C4	112.70 (13)	C16—C14—C13	118.88 (16)
O3—C3—H3	108.8	C16—C14—C15	120.84 (16)
C2—C3—H3	108.8	C13—C14—C15	120.28 (17)
C4—C3—H3	108.8	C14—C15—H15A	109.5
N1—C4—C5	114.69 (13)	C14—C15—H15B	109.5
N1—C4—C3	107.37 (13)	H15A—C15—H15B	109.5
C5—C4—C3	110.77 (13)	C14—C15—H15C	109.5
N1—C4—H4	107.9	H15A—C15—H15C	109.5
C5—C4—H4	107.9	H15B—C15—H15C	109.5
C3—C4—H4	107.9	C17—C16—C14	120.95 (16)
C6—C5—C10	119.27 (15)	C17—C16—H16	119.5
C6—C5—C4	121.45 (14)	C14—C16—H16	119.5
C10—C5—C4	119.28 (15)	C16—C17—C11	119.45 (16)
C7—C6—C5	120.67 (16)	C16—C17—H17	120.3
C7—C6—H6	119.7	C11—C17—H17	120.3
C5—C6—H6	119.7	C4—N1—S1	120.52 (11)
C6—C7—C8	119.90 (17)	C4—N1—H1	119.8
C6—C7—H7	120.1	S1—N1—H1	119.7
C8—C7—H7	120.1	C2—O2—C1	114.07 (13)
C9—C8—C7	119.72 (16)	C3—O3—H3A	109.5
C9—C8—H8	120.1	O5—S1—O4	120.52 (8)
C7—C8—H8	120.1	O5—S1—N1	106.05 (8)
C8—C9—C10	120.35 (16)	O4—S1—N1	106.78 (8)
C8—C9—H9	119.8	O5—S1—C11	107.57 (8)
C10—C9—H9	119.8	O4—S1—C11	107.16 (8)
C9—C10—C5	120.09 (16)	N1—S1—C11	108.29 (8)
			
O1—C2—C3—O3	−2.1 (2)	C11—C12—C13—C14	−0.3 (3)
O2—C2—C3—O3	−179.60 (13)	C12—C13—C14—C16	0.6 (3)
O1—C2—C3—C4	−122.90 (17)	C12—C13—C14—C15	179.70 (17)
O2—C2—C3—C4	59.56 (18)	C13—C14—C16—C17	−0.6 (3)
O3—C3—C4—N1	−61.99 (16)	C15—C14—C16—C17	−179.69 (18)
C2—C3—C4—N1	58.81 (17)	C14—C16—C17—C11	0.3 (3)
O3—C3—C4—C5	63.93 (17)	C12—C11—C17—C16	0.1 (3)
C2—C3—C4—C5	−175.27 (13)	S1—C11—C17—C16	−178.00 (13)
N1—C4—C5—C6	40.6 (2)	C5—C4—N1—S1	72.77 (17)
C3—C4—C5—C6	−81.10 (19)	C3—C4—N1—S1	−163.68 (11)
N1—C4—C5—C10	−139.84 (16)	O1—C2—O2—C1	−1.1 (2)
C3—C4—C5—C10	98.44 (18)	C3—C2—O2—C1	176.38 (13)
C10—C5—C6—C7	0.6 (3)	C4—N1—S1—O5	170.37 (12)
C4—C5—C6—C7	−179.90 (15)	C4—N1—S1—O4	40.68 (14)
C5—C6—C7—C8	−0.8 (3)	C4—N1—S1—C11	−74.42 (14)
C6—C7—C8—C9	0.5 (3)	C17—C11—S1—O5	−147.69 (14)
C7—C8—C9—C10	0.0 (3)	C12—C11—S1—O5	34.16 (16)
C8—C9—C10—C5	−0.2 (3)	C17—C11—S1—O4	−16.75 (16)
C6—C5—C10—C9	−0.1 (2)	C12—C11—S1—O4	165.10 (13)
C4—C5—C10—C9	−179.64 (15)	C17—C11—S1—N1	98.09 (15)
C17—C11—C12—C13	−0.1 (2)	C12—C11—S1—N1	−80.05 (14)
S1—C11—C12—C13	178.01 (13)		

### Hydrogen-bond geometry (Å, °)

**Table:**

*D*—H···*A*	*D*—H	H···*A*	*D*···*A*	*D*—H···*A*
O3—H3A···O2^i^	0.84	2.50	3.270 (2)	152
C1—H1C···O1^ii^	0.98	2.52	3.392 (2)	149
C4—H4···O3^iii^	1.00	2.50	3.484 (2)	166
C1—H1C···O1^ii^	0.98	2.52	3.392 (2)	149

Symmetry codes: (i) *x*, *y*+1, *z*; (ii) −*x*+1, −*y*+1, −*z*+2; (iii) *x*, *y*−1, *z*.
